# A small step or a giant leap: Accounting for settlement delay and dispersal in restoration planning

**DOI:** 10.1371/journal.pone.0256369

**Published:** 2021-08-18

**Authors:** Ana Rodriguez-Perez, Mark A. James, William G. Sanderson

**Affiliations:** 1 Centre for Marine Biodiversity & Biotechnology, EGIS, Heriot-Watt University, Edinburgh, United Kingdom; 2 Scottish Oceans Institute, University of St. Andrews, St Andrews, United Kingdom; 3 St Abbs Marine Station, St Abbs, Scottish Borders, United Kingdom; Bigelow Laboratory for Ocean Sciences, UNITED STATES

## Abstract

Understanding larval duration and hence dispersal potential of the European oyster *Ostrea edulis* is crucial to inform restoration strategies. Laval duration has an obligatory period of maturity to pediveliger (when larvae are ready to settle), but also an unknown period until metamorphosis is triggered by a settlement cue. The extent to which larvae can prolong the pediveliger period and delay metamorphosis has not been studied. Here we show that *O*. *edulis* larvae can delay metamorphosis for a period of 11 days, while retaining the capability to settle in high proportions when presented with a suitable settlement cue. *O*. *edulis* larvae are likely to be able to delay metamorphosis even further, since 80% of larvae in the control treatment were still alive when the experiment was terminated at day 14. The results indicate the ability of *O*. *edulis* larvae to more than double pelagic duration and probably further delay metamorphosis. We discuss these findings in the context of larval mortality, and the importance of *O*. *edulis’* larval settlement requirements for dispersal potential, recruitment success and connectivity of restoration sites.

## 1. Introduction

Ecosystem restoration is increasingly being recognised as a pressing need for counteracting the environmental emergency we are facing. The UN has officially proclaimed the decade of 2021–2030 as the Decade on Ecosystem Restoration following a proposal for action of over 70 countries. Restoring biodiversity and ecosystem services is also a priority in Europe under the European Green Deal and the Biodiversity Strategy to 2030, where the development of legally binding EU nature restoration targets is a key commitment [1]. As ecosystem engineers, oyster reefs and beds play a critical ecological role and provide important ecosystem services [2, 3], yet they are also one of the most imperilled marine habitats on Earth [4]. Restoring oyster habitats has therefore emerged as a priority in many countries around the world, particularly in Europe, the USA and Australia.

In Europe, efforts are mounting to restore the European oyster *Ostrea edulis* and thereby contribute to national and international commitments. *O*. *edulis* once formed widespread beds along European coastlines [5], which were biodiversity hotspots in an otherwise sedimentary environment [6–8]. Former *O*. *edulis* beds likely had an important effect on water purification, light penetration and sediment stabilisation [2, 3, 9–11] and they were an essential part of food security [12, 13]. The natural distributional range of *O*. *edulis* extends from the western European coast of the Norwegian Sea to the Atlantic coast of Morocco and into the Mediterranean Sea and the Black Sea [14]. Today *O*. *edulis* has declined to functional extinction in many areas of the NE Atlantic following heavy fishing in the late 1800s and early 1900s [13, 15–17]. *O*. *edulis* is now internationally recognised as ‘threatened and declining’ in the North-East Atlantic [18]. There is consequently substantial international interest in restoring populations throughout the former range to recover ecological function [13, 19–22], with currently 19 *O*. *edulis* restoration projects in Europe collaborating under the umbrella of the Native Oyster Restoration Alliance (NORA) [21, 22]. In many cases, restoration also requires the recreation of lost shell habitat with an opaque understanding of the exact former locations [20].

Benthic marine invertebrates, such as oysters, rely on pelagic larval recruitment for their populations to persist. This can be either self-recruitment, i.e. the larvae settles in the parental population, or recruitment of larvae originating from geographically distant populations [23]. How much of each type of recruitment occurs defines the amount of gene flow and connectivity within meta-populations. The two fundamental questions to be answered are: where do larvae come from (i.e. the source populations of settling larvae) and where do they go to (i.e. the settlement sites or sink locations of dispersing larvae) [24, 25]. To answer both questions it is critical to understand pelagic larval dispersal–i.e. the time larvae stay in the water before settlement. Pelagic larval duration is the most widely used proxy of dispersal potential in marine species [26–28] and thus a fundamental component in the study of population connectivity [29, 30]. Understanding larval dispersal is therefore key for ensuring the persistence and connectivity of restored *O*. *edulis* beds, as well as to inform where to restore new populations to ensure connectivity and recruitment [31–33].

**Fig 1:**
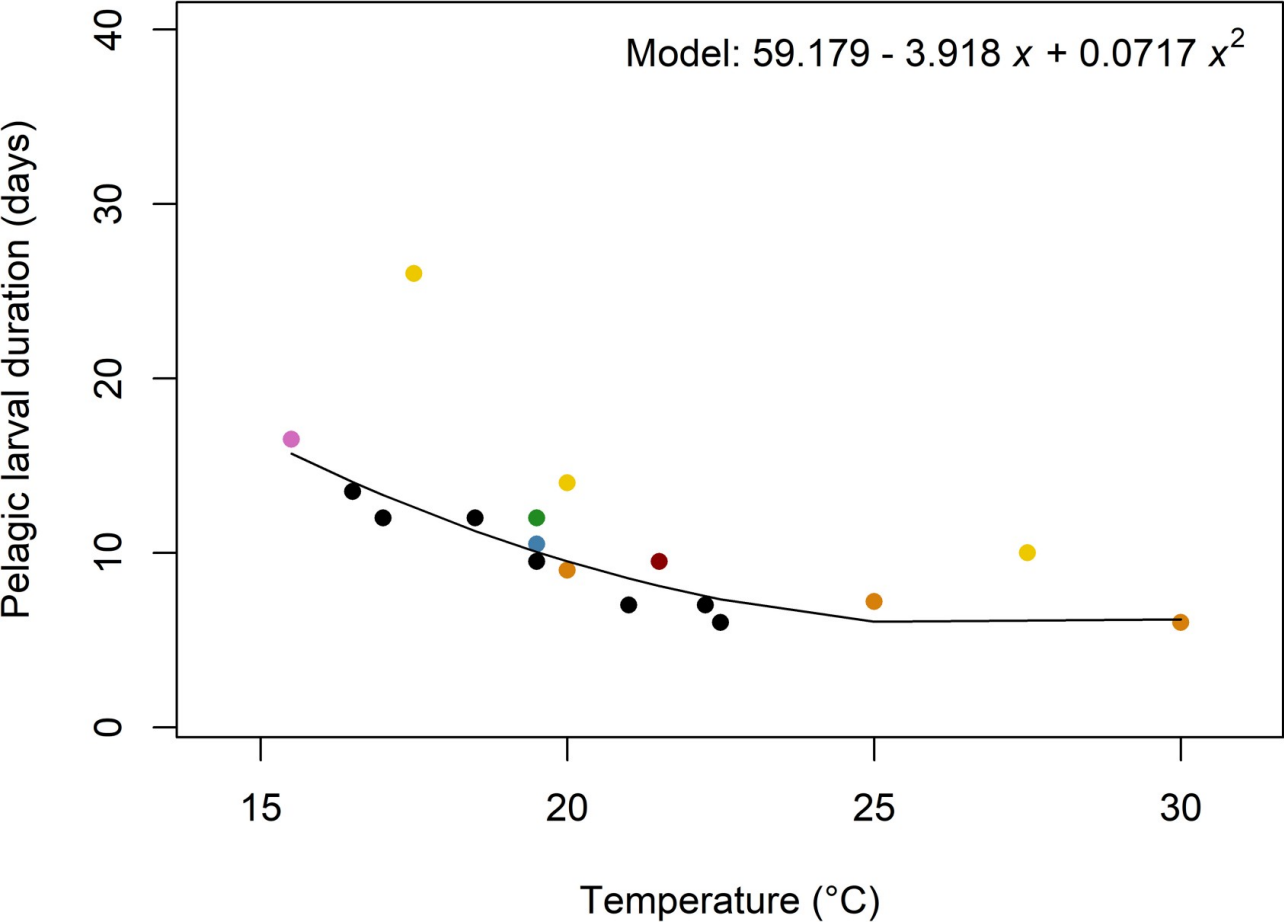
Pelagic larval durations described for *O*. *edulis* larvae in the literature with fitted model. Black points = field data; coloured points = laboratory data, with each colour denoting a different source; yellow points = varying food quality in the laboratory prolonged development time. Median values were plotted when ranges where given (see Table A1 in S1 Appendix for original values and data sources). Only data points where < 50% of larvae were reported to be at pediveliger stage were plotted. Black line shows fitted model: pelagic larval duration in days = 59.179–3.918 *x* + 0.0717 *x*^2^, where *x* is temperature in °C (see Table A2 in S1 Appendix for more details). Data points in which food quality varied (yellow colour) were excluded from the model.

Pelagic larval duration is composed of two components: a period of obligatory dispersal before larvae become competent to metamorphose, and a possible extension of that period until metamorphosis is triggered by a cue [34, 35]. The period of obligatory dispersal can last minutes to days in lecithotrophic larvae (which obtain energy from yolk reserves) and days to weeks for planktotrophic larvae (which feed on plankton) [35]. Obligatory dispersal time can also vary considerably within a single species [36, 37], because larval development is strongly affected by temperature and food [38–40]. Inadequate food quantity and/or quality significantly delays development [40, 41] and higher temperatures result in faster larval development [37, 41]. The population of a species at lower latitudes will therefore have a shorter pelagic duration than a population occurring at higher latitudes [39]. Pelagic duration of planktotrophic *O*. *edulis* larvae described in the literature range from 6 days at 30˚C (in the laboratory) and 6 days at 22˚C (in the Dutch Oosterschelde) to 16–17 days at 15–16˚C and 26 days at 17.5˚C, when variable food quality prolonged development [37, 41, 42] (Fig 1; Table A1 in S1 Appendix).

To date, pelagic duration of *O*. *edulis* larvae has been studied mainly to enhance aquaculture production. Therefore, a possible extension of the pelagic duration period once larvae are competent to settle has not been well considered. Larval duration can be prolonged if suitable settlement sites are absent: metamorphosis is delayed, while maintaining the capacity to metamorphose [34]. The capability of competent larvae to delay metamorphosis in the absence of adequate cues provides an opportunity to be transported to a more suitable habitat for survival and reproduction [34]. *O*. *edulis* larvae appear to be selective settlers, finely-tuned to settle in response to specific substrates [43–45] and cues which are indicative of their adult habitat requirements, such as conspecifics or habitat-associated biofilms [31, 44]. Evidence suggest that *O*. *edulis* larvae can delay metamorphosis for a couple of days [31, 46], but the extent to which larvae are capable of delaying metamorphosis has not been studied.

Biophysical modelling, i.e. combining hydrodynamic models with particle tracking models, which mimic biological traits, has emerged as a powerful tool to simulate and predict larval dispersal. Pelagic larval duration is arguably the most important biological variable that can be included in the biophysical models, as it determines the duration that larvae will be subject to the transport of oceanic currents, a key factor controlling larval dispersal [27, 29, 47]. Biophysical models have been used to simulate larval dispersal and inform conservation measures in several bivalve species. For instance, in Pamlico Sound (USA) for the Eastern oyster *Crassosstrea virginica* [47], in Strangford Lough (Ireland) for *O*. *edulis* [48], and in the Irish Sea for the common cockle *Cerastoderma edule* [49] and the horse mussel *Modiolus modiolus* [50]. The ambition of current *O*. *edulis* restoration and conservation efforts is to be able to predict larval dispersal with biophysical models [e.g. 48]. There is, however, a knowledge gap on the extent to which *O*. *edulis* larvae can delay metamorphosis once they are competent to settle. Understanding the extent to which delayed metamorphosis can influence pelagic duration will also contribute to answering one of the top 40 questions most important to the policy and practice of native oyster reef restoration in Europe [51] i.e. “How can a map of the connectivity potential of restoration sites (accounting for current populations) be developed?” Since the success of oyster populations is often limited by poor understanding of site-specific dispersal patterns of larvae [48], increasing our understanding of pelagic larval duration and the factors affecting metamorphosis will be crucial to correctly parametrise larval dispersal models and ultimately inform conservation and restoration planning.

The aim of the present research was to study the extent to which *O*. *edulis* larvae can delay metamorphosis and thereby influence pelagic duration, dispersal potential and recruitment success, with the goal of informing conservation and restoration efforts. For this purpose, the larvae’s ability to delay metamorphosis once competent to settle, while maintaining the capacity to metamorphose and survive as a spat, was examined.

## 2. Methods

Larvae were obtained from adult *Ostrea edulis* originating from the Danish Limfjord. Adult *O*. *edulis* oysters were conditioned to spawn at the Danish Shellfish Centre according to the FAO guidelines [52]: their holding tank temperature was raised daily by 1–1.5˚C up to 21˚C, after which the temperature was maintained at 20–22˚C. Adult oysters were fed daily with a mixture of *Chaetoceros muelleri*, *Rhodomonas salina*, *Tisochrysis lutea* and *Pavlova gyrans* (volume ratio 20:7:1:1 respectively). Newly spawned *O*. *edulis* larvae were transferred into 15 L flow-through holding tanks with 1 μm filtered seawater (FSW) at 25˚C. Larvae were raised at concentrations of 10 larvae/ml and fed daily a microalgae mixture consisting of *Chaetoceros muelleri*, *Tisochrisys lutea* and *Pavlova gyrans* (volume ratio 5:1:1) at a concentration of *circa* 100 cells/μl. Every 1–2 days larvae were monitored with a binocular microscope for developmental stage. After 9 days, 60% had developed to pediveliger stage, in which larvae are mature to settle.

**Fig 2:**
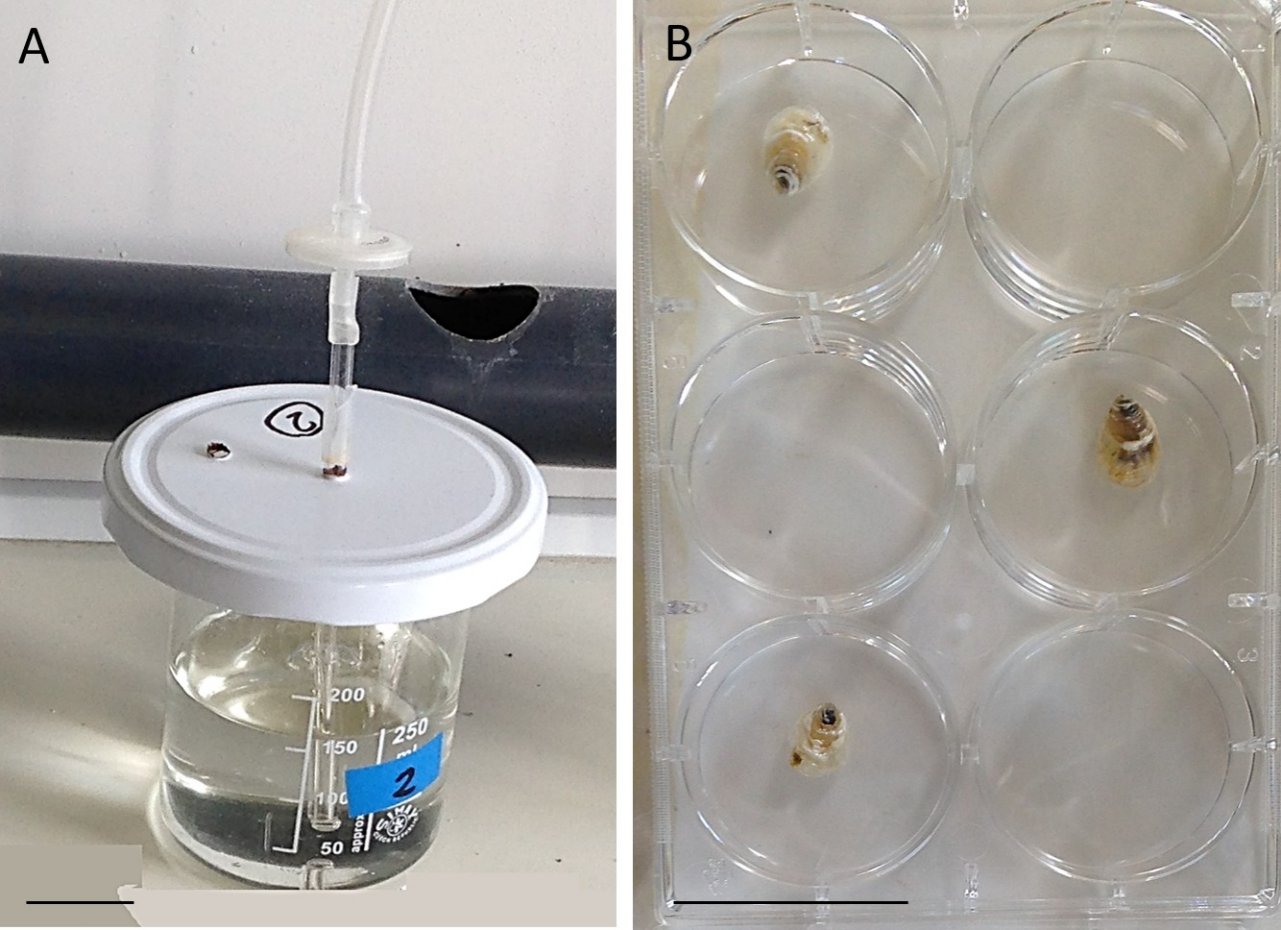
A) One of three culture beakers (250 ml, fitted with 0.2 μm-filtered aeration) with pediveliger larvae for experimentation. B) Example of experimental well plate used in the settlement tests with *O*. *edulis* spats for the ‘highly suitable’ treatment (see Table 1 for specifications of the two treatments). Scale bars: 3 cm.

On day 9, a subsample of larvae was examined under a binocular microscope, to selectively pipette pediveliger for experimentation (N = 153). Selected larvae were divided into three 250 ml beakers at a concentration of 0.4 larvae/ml. The beakers were covered by a lid and fitted with slow aeration filtered to 0.2 μm (Fig 2A). Larvae were maintained in 0.2 μm FSW at an average (± SE) room temperature of 24.2 ±0.01˚C and continued to be fed according to the schedule above. The beakers were maintained and monitored for the duration of the experiment: every 3 days the beaker water was changed [52] and dead or settled larvae were counted and removed during water changes.

**Table 1:** Treatments used to study delay of metamorphosis in *O*. *edulis* larvae.

Treatment	Settlement cue	Description
Highly suitable	Conspecific	1 μm filtered seawater (FSW) with a living *O*. *edulis* spat (length: 1–2 cm) and food (100 cell/μl of *Chaetoceros muelleri*, *Tisochrisys lutea* and *Pavlova gyrans* at a volume ratio of 5:1:1)
Unsuitable	No cue: control	1 μm FSW with food as in treatment above and no spat. Control treatment to account for any effect that water quality parameters could have on the settlement of *O*. *edulis* larvae.

Settlement suitability according to a previous study [31].

**Fig 3:**
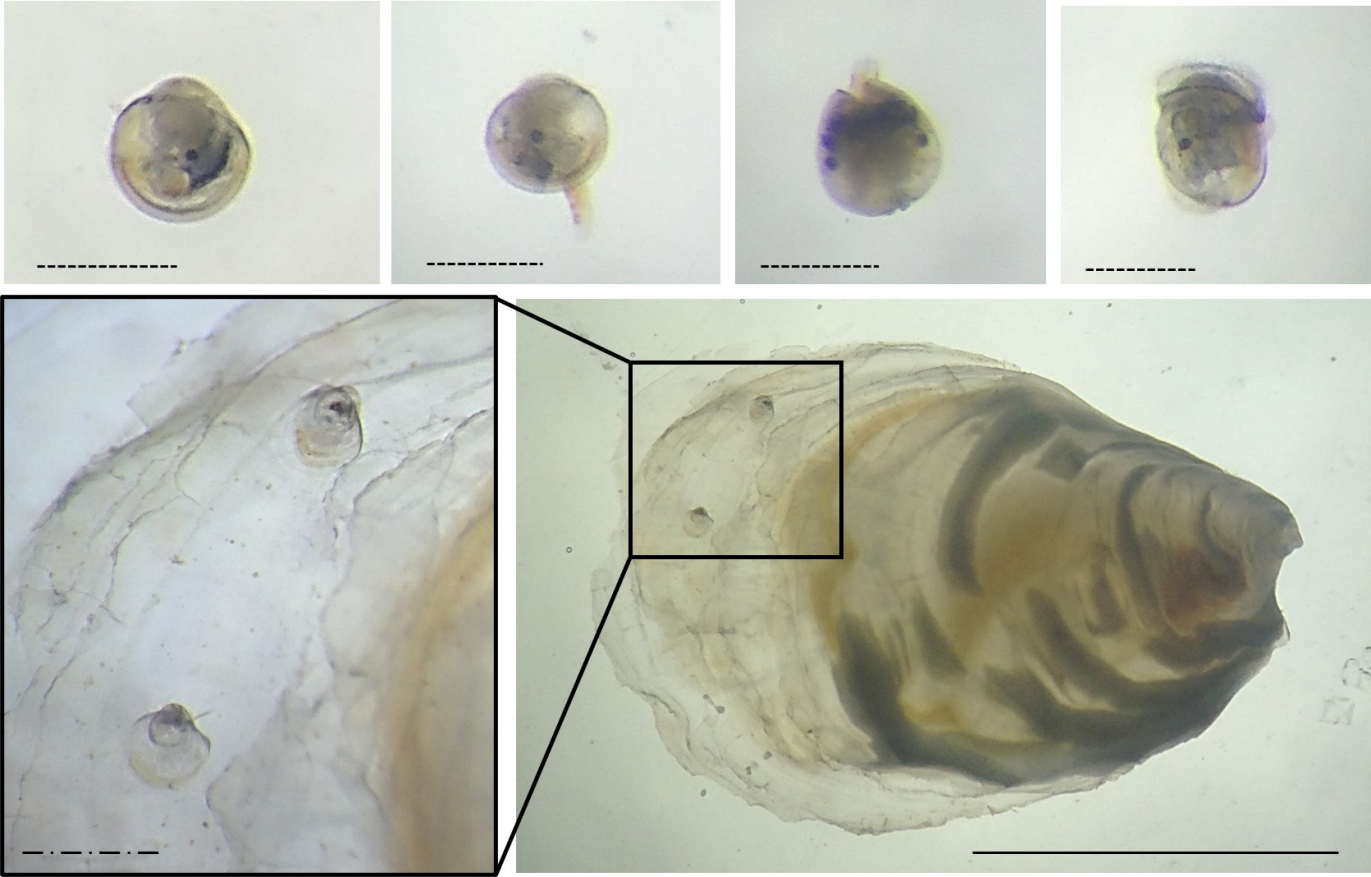
Top row: *O*. *edulis* pediveliger larvae that did not settle (with visible eyespots in all pictures and protruding foot in the central two). Bottom row: *O*. *edulis* spat from the ‘highly suitable’ settlement treatment with two *O*. *edulis* larvae metamorphosed on it. Scale bars: dashed = 300 μm; dashed-dotted = 1 mm; full = 1 cm.

Settlement viability of pediveliger larvae was examined on days 0, 4, 7 and 11 (each called a ‘settlement test’ hereafter). In every settlement test, 41–48 larvae from the beaker culture were subjected to two treatments, which had previously been determined to be ‘highly suitable’ (conspecifics) and ‘unsuitable’ (control sea water) for settlement [31] (Table 1; Fig 2B). Each treatment was replicated six times and randomly allocated to a well in two 6-well culture plates. Four larvae were assigned into each well replicate with 24 larvae per treatment, except for the last settlement test, where remaining larvae (N = 41) where divided between the treatments (five wells with 4 larvae/well and seven with 3 larvae/well). After three days, the status of each larvae (‘alive’/ ‘dead’ and ‘settled’/ ‘not settled’) was examined with a binocular microscope. Living spat were identified by the brownish and yellowish colouration of their organs, particularly around the umbo area (see Fig 3). Dead spat were white-translucent with open valves and dead larvae were identified by a faded colour of their inner organs as well as prolonged immobility and open valves (see Fig 3 for an example of the inner colouration of living larvae). Settled spat were measured and placed into a growing tray and after three days, survival and growth were examined under a binocular microscope. Spat were measured with a measuring ruler of 1mm certified length that had been fitted into the eyepiece of a binocular microscope.

### Statistics

Fisher’s Exact test was used to test for significant differences between the number of living larvae, which had settled or not settled in each settlement test and treatment. Significant differences in mortality per settlement test and treatment were also tested with Fisher’s Exact test. All data analysis was performed in R v.3.4.0 [53].

## 3. Results

**Fig 4:**
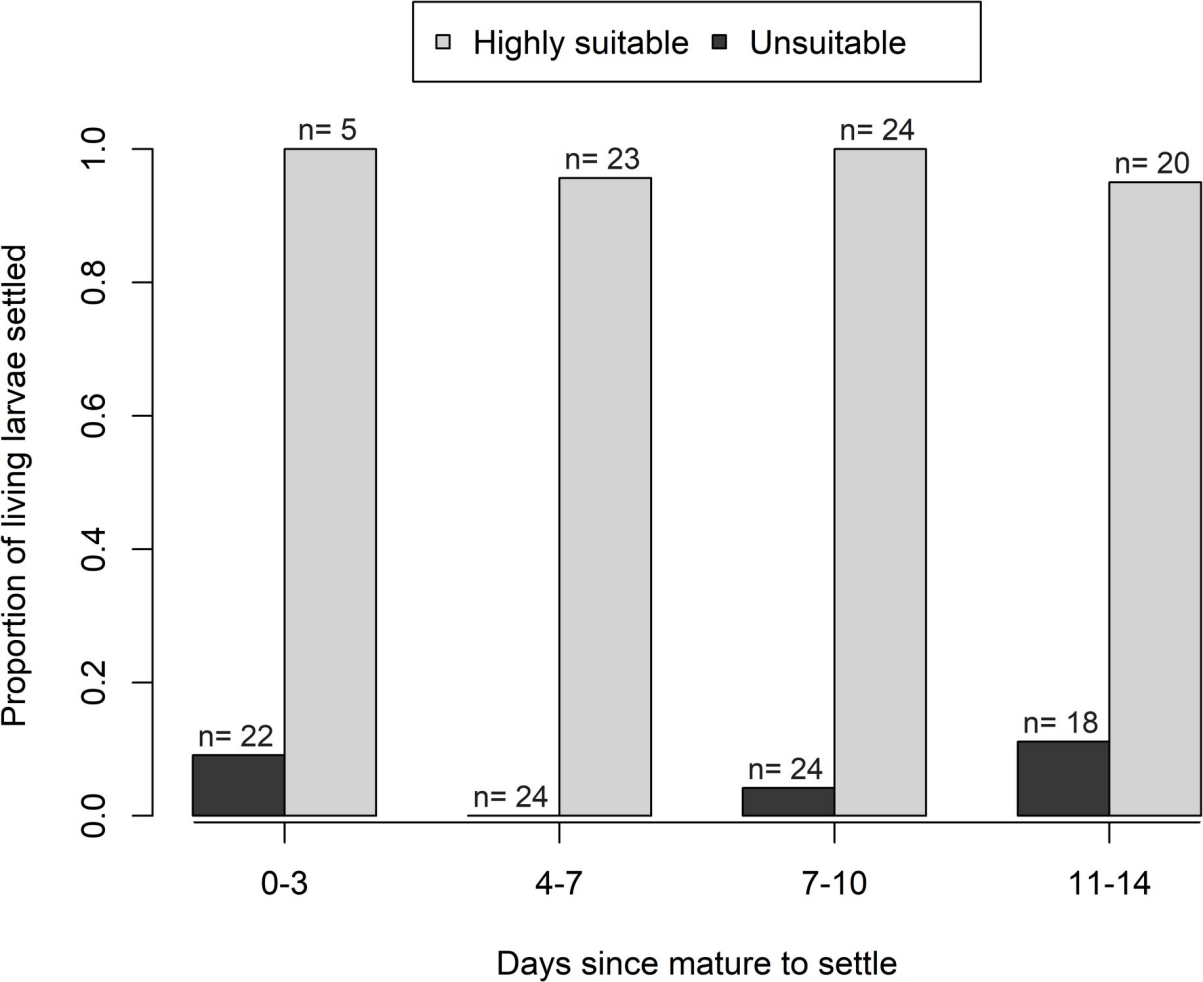
Proportion of pediveliger larvae able to settle with increasing time since maturation. The ‘highly suitable’ settlement treatment contained a spat as the settlement cue whereas the ‘unsuitable’ settlement treatment contained only filtered seawater with food. N is number of larvae that were observed and still alive at the end of each 3-day experimental test (see also Fig 5). All larvae were mature to settle from day 0.

**Fig 5:**
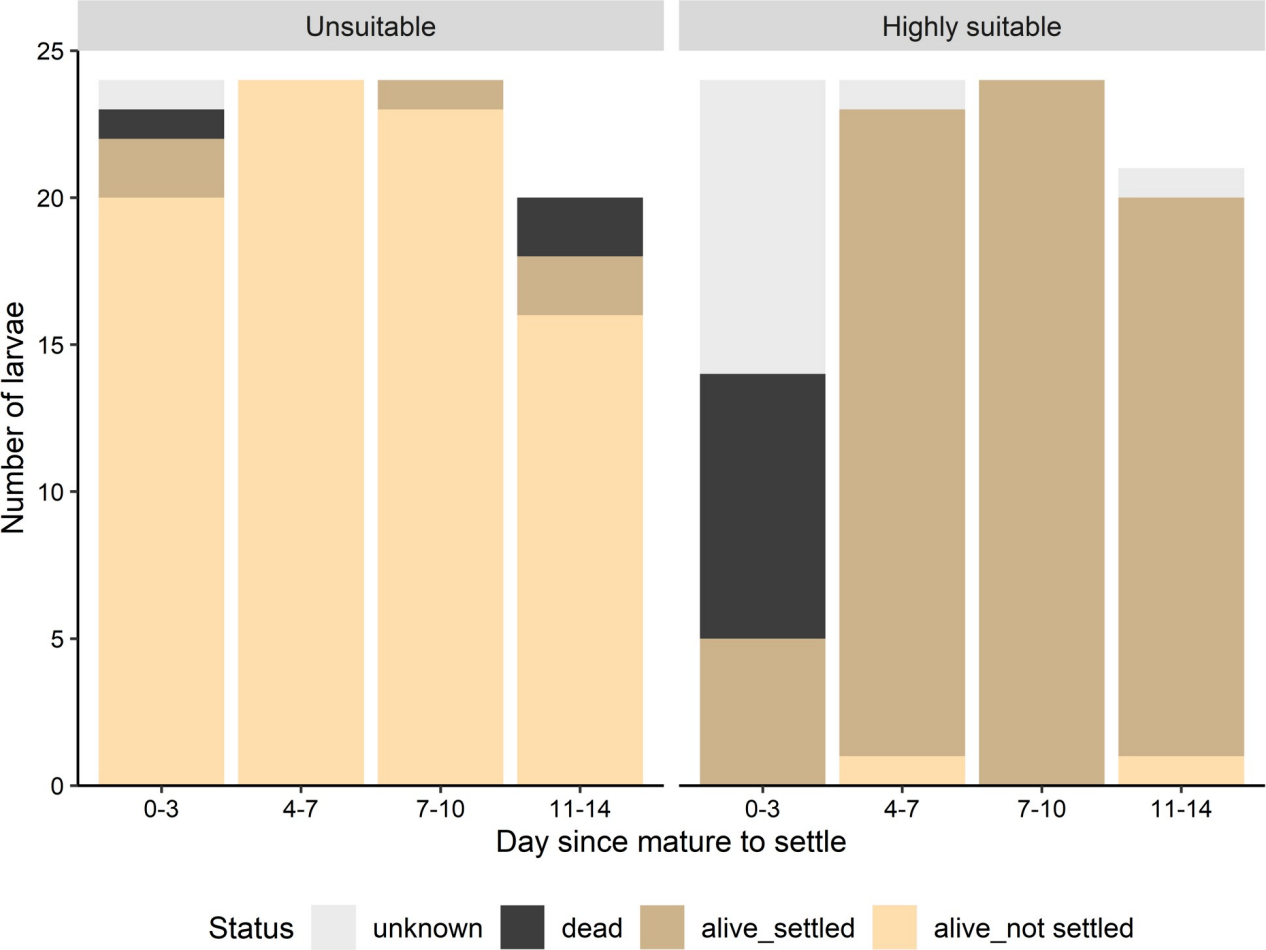
Total number of larvae used per treatment and their respective status after each settlement test: Alive-settled, alive-not settled, dead and unknown (not found). Only ‘not-settled larvae’ were dead, all spat (settled larvae) were alive.

*Ostrea edulis* larvae were able to settle 11 days after reaching pediveliger stage (Fig 3): 95% (19/20) when presented with a ‘highly suitable’ settlement treatment on day 11 metamorphosed successfully compared to 11% (2/18) in the ‘unsuitable’ treatment (Fig 4). 80% of all larvae (16/20) from the ‘unsuitable’ treatment, were still alive and had not metamorphosed 14 days after reaching maturity to settle (pediveliger stage), when the experiment was terminated due to the low number of remaining ‘non-settled’ larvae (N = 17 out of 41) (Fig 5). When comparing live larvae, the number of settled larvae versus not-settled larvae did not differ significantly between settlement tests of larvae at different days post pediveliger in the ‘highly suitable’ treatment (Fisher’s Exact test, df = 3, *p* = 0.60) nor in the ‘unsuitable’ treatment (Fisher’s Exact test, df = 3, *p* = 0.32). Overall, 95–100% of living larvae settled in the ‘highly suitable’ treatment, 0–11% in the ‘unsuitable’ treatment (Fig 4) and 0–9% in the beakers (Fig 2A) with larvae for future settlement tests (Table 2).

**Table 2:** Percent of settled, living and dead larvae in the pediveliger culture beakers (Fig 2A) after each water change.

Days since start	% alive	% dead	% settled	N
0	100.00	0.00	0.00	153
3	97.87	2.13	6.38	94
6	96.23	3.77	9.43	53
7	100.00	0.00	0.00	42
11	100.00	0.00	0.00	21

The number of mortalities (both settled and not settled) was significantly different between settlement tests within the ‘highly suitable’ settlement treatment (Fisher’s Exact test, df = 3, *p* < 0.001) and the ‘unsuitable’ treatment (Fisher’s Exact test, df = 3, *p* = 0.047). In the ‘highly suitable’ treatment, 37.5% (9/24) of larvae died and 58% (14/24) were not found in the first settlement test (day 0–3), while only 4.2% (1/24) died and 4.2% (1/24) were not found in the ‘unsuitable’ treatment (Fig 5). In subsequent settlement tests, all larvae in the ‘unsuitable treatment’ were accounted for and nearly all in the ‘highly suitable’ treatment (Fig 5). No further dead larvae were found in any of the subsequent ‘highly suitable’ treatments. In the ‘unsuitable’ treatment, there were no further dead larvae either until the last settlement test (day 11–14), when 10% of larvae died (2/20; Fig 5). Only non-settled larvae were found dead at the end of each settlement test, all settled spat were alive. Mortality of larvae in the pediveliger culture beakers (Fig 2A) was <4% until the end (day 11, see Table 2).

Survival of settled spat was 96–100% within the three days of monitoring. The spat grew from average ± se length x width of 458 ± 2.9 x 459 ± 3.8 μm to 625 ± 12 x 582 ± 12.9 μm. Spat survival and growth was not compromised with increasing delay of metamorphosis (Table 3).

**Table 3:** Mean size of spat after each settlement test, as well as their growth and survival three days later.

	Day 3	Day 7	Day 10	Day 14
Initial size [μm]	–	–	428 x 381	487 x 510
Size [μm] 3 days later	–	649 x 599	608 x 578	613 x 568
Survival (alive/ total) 3 days later	–	21/22	19/19	19/19

No measurements were taken = ‘–‘.

## 4. Discussion

The aim of this study was to examine the ability of *Ostrea edulis* larvae to delay metamorphosis and hence to increase their larval duration and dispersal potential. *O*. *edulis* larvae were able to delay metamorphosis for at least 11 days under experimental conditions. The proportion of larvae metamorphosing in response to the ‘highly suitable’ treatment (95–100%) did not differ significantly over the 11 days and it was similar to the 100% settlement observed in a previous study in response to conspecifics [31]. Settled spat survival was 96–100% during the three days of monitoring. Spat mortality is highest during the first few days after settlement [54–56], it is therefore likely that the *O*. *edulis* spat would have continued growing healthily, despite a delay in metamorphosis.

At day 14, 80% of larvae (16/20) in the ‘unsuitable’ treatment were still alive and had not settled, indicating that *O*. *edulis* larvae are likely to be able to delay metamorphosis even further. Related bivalve species were able to delay metamorphosis for 28–46 days (blue mussel *Mytilus edulis* [34]) and for at least 30 days (pacific oyster *Crassostrea gigas* [57]) suggesting that *O*. *edulis* may be similar. *M*. *edulis* and *C*. *gigas* larvae have slightly longer pelagic durations than *O*. *edulis* [*cf*. ref [52] for *C*. *gigas* and ref. [58] for *M*. *edulis*] because fertilization of *O*. *edulis* larvae occurs inside the mother oyster and larvae are brooded for about 7–10 days before being released into the water column [37]. However, the overall developmental time from fertilisation to pediveliger was similar for *O*. *edulis* and *C*. *gigas* [52, p.102]. The capacity to delay metamorphosis is thought to depend on the rate of development, which, in turn, depends on seawater temperature and the amount and quality of the food available [39–41]: the longer the pre-competent period, the greater the capacity for delaying metamorphosis [34]. In addition, the amount and quality of the food available during development may also affect the period of competence and proximate post settlement mortality when the metabolic demands of the transition from a planktonic to a sessile life are likely to be most acute. In the present experiment, larvae were fed three microalgae species (*Chaetoceros muelleri*, *Tisochrisys lutea* and *Pavlova gyrans*) according to the hatchery procedures of the Danish Shellfish Centre. This mix of microalgae represents a good food source with fast growth (on average 7 days at 25°C), but under natural conditions the food regime may be more appropriate since *O*. *edulis* larvae develop faster in the sea than in the laboratory experiments (see Fig 1). The quality and quantity of food in the sea is likely to be variable [59] and this may be exacerbated by mismatches in the timing of spawning and larval development with phytoplankton blooms as a result of climate change [60]. Food quality and quantity is of critical importance for larval development to pediveliger [40, 41], but it is not clear to what extent it affects the larvae’s capacity to delay metamorphosis. For instance, *M*. *edulis* larvae’s ability to delay metamorphosis was barely affected by differences in quality and quantity of food [58]. More studies are therefore needed to elucidate the extent to which the feeding regime can affect *O*. *edulis* larval ability to delay metamorphosis.

The period of time larvae delay their metamorphosis is strongly correlated to their specific substrate and habitat requirements [57]. Some species have an extensive target habitat and can settle almost anywhere in the benthos (e.g. the crab *Cancer magister* [27]), whilst others have restricted target habitat and require cues specifically indicative of surfaces where those species occur [61]. Larvae delay metamorphosis if required settlement cues are absent, as well as if they sense the presence of dominant competitors [34] or predators [62]. As metamorphosis is delayed, larvae can become more sensitive to environmental stimuli, which trigger metamorphosis [34, 57]. For instance, in previous experiments, *O*. *edulis* larvae settled preferentially in response to particular substrates [43–45] and immediately in response to conspecifics, but it took them almost two days to start settling in response to a biofilm cue from a natural habitat [31].

When habitat-associated cues were absent in the present and previous studies, the proportion of larvae settling under low larval concentrations (i.e. <2 larvae ml^-1^) was remarkably low and constant both over time and across experiments (Table 4). Larvae metamorphosed consistently at rates <15% despite the presence of hard substrate (shell or stones but lacking an appropriate cue [31]) and despite having delayed metamorphosis for 11 days (present experiment). This indicates that *O*. *edulis* larvae are specialist settlers [*cf*. 61], finely-tuned to target conspecifics and their own habitats [31, 44]. Specialist settlers metamorphose at low rates on surfaces which provide a habitat that is neither optimal nor deleterious for juveniles [63]. This is reflected in the observed low settlement rates of *O*. *edulis* on such surfaces (Table 4).

**Table 4:** Summary of *O*. *edulis* larval settlement in response to treatments, which were neither optimal nor deleterious.

Source	Treatment	Settlement surface	Larval concentration (larvae ml^-1^)	Settlement proportion
Current study	‘Unsuitable’ treatment	6-well culture plate	0.67	0–11%
Current study	Reserve larvae in beaker	Glass surface of Plexiglass beaker (250 ml)	0.4	0–9%
[31]	All treatments, except spat and biofilm	6-well culture plates with (i) oyster shell, (ii) stone, (ii) microalgae food or (iv) without any added material/food	0.67	0–14%
[64], p.82	Larvae in holding tanks	Glass surface of aquarium (3 litres)	1.5	0–9%

The benefits of being able to delay metamorphosis are clear: it increases the larvae’s chance of finding a suitable settlement location, which will support survival and reproduction [34]. However, as larvae delay metamorphosis, the risk of predation in the water column increases [37], and if larvae do not find a suitable settlement location, many may eventually die without metamorphosing [57, 65, 66]. In the sea, the largest proportion of *O*. *edulis* larvae may therefore be lost through mortality if their settlement preferences are not fulfilled.

In this study there were significant differences in mortality between both treatments. During the first settlement test (day 0–3), many larvae died in the ‘highly suitable’ spat treatment (38%), but not in the control treatment (4%). This indicates contamination originating from the spat (e.g. their faeces resulting in increased bacterial growth), which may have been exacerbated by warm temperatures (~24°C). In addition, a large proportion of larvae from the ‘highly suitable’ treatment (58%) were unaccounted for at the end of that settlement test. These larvae may have also died and subsequently been ingested by the spat through its filtering-feeding behaviour. No further mortality was observed until day 14, when 10% of control larvae died. Natural mortality was thus low throughout the 14 days of observation. However, if the experiment had continued, *O*. *edulis* larvae would presumably have eventually died or metamorphosed spontaneously, with metamorphosis of *O*. *edulis* larvae being able to occur in response to chemical stimuli [67] and without the need of prior attachment to a substrate (personal observation at Danish Shellfish Centre). Future experiments may investigate where that balance lies, and whether, in the absence of suitable settlement cues, most *O*. *edulis* larvae would eventually die (despite the absence of predation), or whether they would spontaneously metamorphose.

Pelagic larval duration is the most widely used proxy for dispersal potential. However, while a species with short pelagic duration will inevitably have a short dispersal, species with long pelagic duration do not necessarily disperse more widely [27]. This is because larval behaviour can contribute to retention or return to natal sites with their behaviours [68, 69]; thus breaking the otherwise direct relationship between pelagic larval duration and dispersal distance [25]. In the laboratory, *O*. *edulis* larvae displayed a strong benthic preference throughout their development [32]. If this behaviour is not overridden by local hydrodynamics [33], it would markedly reduce dispersal distances despite potentially long development times. This study showed that larval behaviour can also considerably prolong pelagic durations if settlement preferences are not met. Potential dispersal time was increased by 2.2-fold in the present study: from 9 days, in which larvae developed to pediveliger stage, to an additional 11 days, in which larvae delayed metamorphosis due to a lack of suitable settlement cues.

The selectivity of *O*. *edulis* larvae at settlement indicates that there is a need to better understand the settlement cues, their efficiency, and the manner in which they are dispersed and sensed to increase restoration success. For instance, if the primary settlement cue is chemical and derived from conspecifics, do beds have to be of a minimum critical size/concentration of living oyster biomass to release sufficient cue to trigger settlement effectively in an open sea environment? What is the strength of the cue of a spat versus an adult oyster in the open sea: would it be possible to lay spat-on-shell on top of dead cultch material to increase settlement success? Alternatively, would it be possible to identify the cue, synthesize it and release it in association with the provision of dead cultch material to increase natural settlement in the initial stages of restoration? These are questions that could be investigated further as they have the potential to significantly increase settlement and thus restoration success.

In conclusion, pelagic duration of *O*. *edulis* larvae–and hence its dispersal potential–can vary considerably. Obligatory development times last from 6 to >20 days (Fig 1), but once competent to settle, larvae can prolong their pelagic duration for at least 11 days, and probably longer. Larvae can therefore remain pelagic for considerable time, if suitable settlement sites are absent, and this is likely to also depend on seawater temperature and the abundance and quality of phytoplanktonic food. For this reason, connectivity of oyster restoration sites may be greater at greater distances than might otherwise be expected. Conversely, prolonged pelagic larval duration also increases larval mortality, which can strongly diminish recruitment strength. In this study the potential dispersal time was more than doubled through the delay in metamorphosis, indicating the important role that the larvae’s settlement requirements can play when planning for dispersal potential, habitat connectivity and recruitment success in restoration programmes.

## Supporting information

S1 Appendix (DOCX)
