# Supplementary material for: A small step or a giant leap: Accounting for settlement delay and dispersal in restoration planning
Source: PLoS One. 2021 Aug 18;16(8):e0256369. doi: 10.1371/journal.pone.0256369 (PMC8372959; doi:10.1371/journal.pone.0256369)
Supplement: S1 Appendix — (DOCX) [file pone.0256369.s001.docx]

S1 APPENDIX

**Table A1.** Review of development time (days) with temperature of *O. edulis* larvae from swarming to mature pediveliger (≥ 50% pediveliger, except where otherwise specified). Sources marked with an asterisk are cited in reference 37.

| Temperature (°C) | Development time (days) | Source | Data | Comments |
| --- | --- | --- | --- | --- |
| 15 | 12 | [41] | Laboratory | 11.7% pediveliger |
| 15-16 | 16-17 | Mazzarelli (1922)* | Laboratory |  |
| 16-17 | 13-14 | [37] | Field | Larval setting observed |
| 17 | 12 | [37] | Field | Larval setting observed |
| 17.5 | 26 | [42] | Laboratory | Food quality varied |
| 18-19 | 12 | [37] | Field | Larval setting observed |
| 18-21 | 9-10 | [37] | Field | Larval setting observed |
| 18-21 | 10-14 | Hagmeier (1916)* | Laboratory |  |
| 19-20 | 10-11 | Cole (1936)* | Laboratory |  |
| 20 | 9 | [41] | Laboratory | 51.7% pediveliger |
| 20 | 14 | [42] | Laboratory | Food quality varied |
| 21 | 7 | [37] | Field | Larval setting observed |
| 21-22 | 9-10 | Cole (1939)* | Laboratory |  |
| 22-22.5 | 7 | [37] | Field | Larval setting observed |
| 22-23 | 6 | [37] | Field | Larval setting observed |
| 25 | 7.2 | [41] | Laboratory | 89% pediveliger |
| 27-30 | 8-12 | [42] | Laboratory | Food quality varied |
| 30 | 6 | [41] | Laboratory | 96% pediveliger |

**Table A2.** Polynomial model fitted to development times (days) of *O. edulis* larvae with temperature data (see Table A1).

| Coefficients | Estimate | Std. Error | t-value | *P* |
| --- | --- | --- | --- | --- |
| Intercept | 59.1792 | 8.776 | 6.743 | **< 0.001***** |
| *x* | -3.9178 | 0.794 | -4.933 | **< 0.001***** |
| *x*^2^ | 0.07172 | 0.018 | 4.083 | **< 0.01**** |
